# (*E*)-1,1,4,4-Tetra­phenyl­but-2-yne-1,4-diol

**DOI:** 10.1107/S160053681000629X

**Published:** 2010-02-20

**Authors:** J. Kalyana Sundar, K. Mohan Kumar, V. Vijayakumar, J. Suresh, S. Natarajan, P. L. Nilantha Lakshman

**Affiliations:** a Department of Physics, Madurai Kamaraj University, Madurai 625 021, India; b Environmental and Analytical Division, School of Advanced Sciences, VIT University, Vellore 632 104, India; c Organic Chemistry Division, School of Advanced Sciences, VIT University, Vellore 632 104, India; d Department of Physics, The Madura College, Madurai 625 011, India; e Department of Food Science and Technology, University of Ruhuna, Mapalana, Kamburupitiya 81100, Sri Lanka

## Abstract

The mol­ecule of the title compound, C_28_H_22_O_2_, is centrosymmetric with the inversion centre located at the mid-point of the C C bond [1.178 (5) Å]. The hydroxyl groups therefore lie on either side of the mol­ecule. The crystal structure is stabilized by O—H⋯O hydrogen bonds, leading to the formation of a linear supra­molecular chain along the *b* axis.

## Related literature

For related structures, see: Braga *et al.* (1997 ▶); Steiner (1996 ▶).
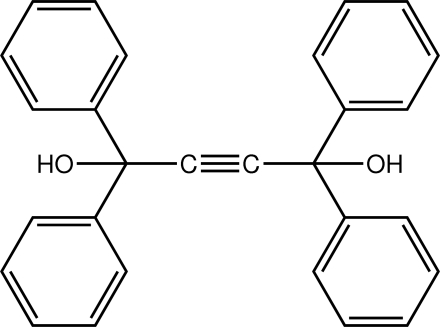


## Experimental

### 

#### Crystal data

C_28_H_22_O_2_
*M* *_r_* = 390.46
Monoclinic, 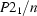

*a* = 11.7760 (7) Å
*b* = 6.1154 (4) Å
*c* = 14.7620 (9) Å
β = 104.930 (8)°
*V* = 1027.20 (11) Å^3^
*Z* = 2
Mo *K*α radiation
μ = 0.08 mm^−1^
*T* = 293 K
0.23 × 0.21 × 0.19 mm

#### Data collection

Nonius Mach3 diffractometer
Absorption correction: ψ scan (North *et al.*, 1968 ▶) *T*_min_ = 0.982, *T*_max_ = 0.985
2268 measured reflections
1793 independent reflections
1190 reflections with *I* > 2σ(*I*)
*R*_int_ = 0.019
2 standard reflections every 60 min  intensity decay: none

#### Refinement

*R*[*F* ^2^ > 2σ(*F* ^2^)] = 0.052
*wR*(*F* ^2^) = 0.276
*S* = 1.09
1793 reflections
136 parameters
H-atom parameters constrained
Δρ_max_ = 0.35 e Å^−3^
Δρ_min_ = −0.29 e Å^−3^

### 

Data collection: *CAD-4 EXPRESS* (Enraf–Nonius, 1994 ▶); cell refinement: *CAD-4 EXPRESS*; data reduction: *XCAD4* (Harms & Wocadlo, 1996 ▶); program(s) used to solve structure: *SHELXS97* (Sheldrick, 2008 ▶); program(s) used to refine structure: *SHELXL97* (Sheldrick, 2008 ▶); molecular graphics: *PLATON* (Spek, 2009 ▶); software used to prepare material for publication: *SHELXL97*.

## Supplementary Material

Crystal structure: contains datablocks global, I. DOI: 10.1107/S160053681000629X/tk2631sup1.cif

Structure factors: contains datablocks I. DOI: 10.1107/S160053681000629X/tk2631Isup2.hkl

Additional supplementary materials:  crystallographic information; 3D view; checkCIF report

## Figures and Tables

**Table 1:** Hydrogen-bond geometry (Å, °)

*D*—H⋯*A*	*D*—H	H⋯*A*	*D*⋯*A*	*D*—H⋯*A*
O1—H1a⋯O1^i^	0.82	2.37	3.040 (3)	139

Symmetry code: (i) 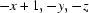.

**Fig. 1.**
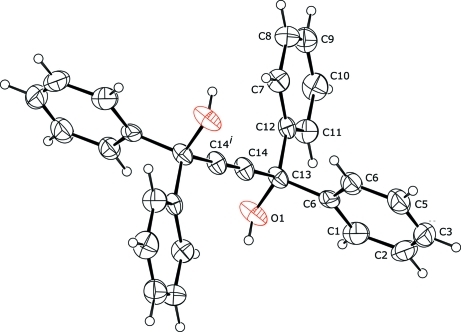
The molecular structure of the title compound, showing 30% probability displacement ellipsoids and the atom-numbering scheme. Symmetry operation i: 1-x, 1-y, -z.

## supplementary crystallographic information

### Comment

As part of our investigations on but-2-yne 1,4-diol molecules, the title molecule, (I), has been synthesized and structurally characterized. The molecule is centrosymmetric with the centre of inversion located at the mid-point of the C14≡C14^i^ bond, Fig. 1; symmetry operation i: 1-x, 1-y, -z. From symmetry, the hydroxyl groups lie on opposite sides of the molecule. The C14≡C14^i^ bond distance of 1.178 (5) Å is comparable with those in uncoordinated alkyne, i.e. 1.193 (3) A° (Braga *et al.*, 1997), and 1.200 (4) Å in 2-butyne-1,4-diol (Steiner, 1996). The OH groups in (I) are engaged in intermolecular hydrogen bonding interactions (Table 1) that lead to the formation of a linear supramolecular chain along the *b* axis.

### Experimental

Sodium acetylide (2.5 ml, 18 wt%, 0.01 M) was placed in a round bottom flask, washed twice with dry THF to remove xylene and light mineral oil. A solution of benzophenone (1.82 g, 0.01 M) in dry THF (10 ml) was added drop-wise to the above mixture and stirred for 2 h. A slight excess of powdered ammonium chloride (5 g) was added gradually to decompose the sodium derivative. The mixture was allowed to stand overnight with stirring (to remove excess ammonia). The residue was extracted with dry THF, the organic layer was washed successively with water; dilute sulphuric acid and sodium hydrogen carbonate solutions, and then dried over magnesium sulphate. The obtained product was purified using column chromatography with hexane and ethylacetate (3:2). The obtained product was recrystallized from diethyl ether. M.pt.: 459–461 K, Yield: 52%.

### Refinement

The H atoms were placed in their calculated positions and allowed to ride on their carrier atoms with C—H = 0.93 Å and O—H = 0.82 Å, and with *U*_iso_ = 1.2*U*_eq_(C) and *U*_iso_ = 1.5*U*_eq_(O) for OH group.

### Crystal data

**Table:**

C_28_H_22_O_2_	*F*(000) = 412
*M**_r_* = 390.46	*D*_x_ = 1.262 Mg m^−^^3^
Monoclinic, *P*2_1_/*n*	Mo *K*α radiation, λ = 0.71073 Å
Hall symbol: -P 2yn	Cell parameters from 25 reflections
*a* = 11.7760 (7) Å	θ = 2–25°
*b* = 6.1154 (4) Å	µ = 0.08 mm^−^^1^
*c* = 14.7620 (9) Å	*T* = 293 K
β = 104.930 (8)°	Block, colourless
*V* = 1027.20 (11) Å^3^	0.23 × 0.21 × 0.19 mm
*Z* = 2	

### Data collection

**Table:**

Nonius Mach3 diffractometer	1190 reflections with *I* > 2σ(*I*)
Radiation source: fine-focus sealed tube	*R*_int_ = 0.019
graphite	θ_max_ = 25.0°, θ_min_ = 2.6°
ω–2θ scans	*h* = 0→13
Absorption correction: ψ scan (North *et al.*, 1968)	*k* = −1→7
*T*_min_ = 0.982, *T*_max_ = 0.985	*l* = −17→16
2268 measured reflections	2 standard reflections every 60 min
1793 independent reflections	intensity decay: none

### Refinement

**Table:**

Refinement on *F*^2^	Primary atom site location: structure-invariant direct methods
Least-squares matrix: full	Secondary atom site location: difference Fourier map
*R*[*F*^2^ > 2σ(*F*^2^)] = 0.052	Hydrogen site location: inferred from neighbouring sites
*wR*(*F*^2^) = 0.276	H-atom parameters constrained
*S* = 1.09	*w* = 1/[σ^2^(*F*_o_^2^) + (0.191*P*)^2^ + 0.1322*P*] where *P* = (*F*_o_^2^ + 2*F*_c_^2^)/3
1793 reflections	(Δ/σ)_max_ < 0.001
136 parameters	Δρ_max_ = 0.35 e Å^−^^3^
0 restraints	Δρ_min_ = −0.29 e Å^−^^3^

### Special details

**Table:**

Geometry. All esds (except the esd in the dihedral angle between two l.s. planes) are estimated using the full covariance matrix. The cell esds are taken into account individually in the estimation of esds in distances, angles and torsion angles; correlations between esds in cell parameters are only used when they are defined by crystal symmetry. An approximate (isotropic) treatment of cell esds is used for estimating esds involving l.s. planes.
Refinement. Refinement of *F*^2^ against ALL reflections. The weighted *R*-factor wR and goodness of fit *S* are based on *F*^2^, conventional *R*-factors *R* are based on *F*, with *F* set to zero for negative *F*^2^. The threshold expression of *F*^2^ > σ(*F*^2^) is used only for calculating *R*-factors(gt) etc. and is not relevant to the choice of reflections for refinement. *R*-factors based on *F*^2^ are statistically about twice as large as those based on *F*, and *R*- factors based on ALL data will be even larger.

### Fractional atomic coordinates and isotropic or equivalent isotropic displacement parameters (Å^2^)

**Table:**

	*x*	*y*	*z*	*U*_iso_*/*U*_eq_	
C14	0.4922 (3)	0.4489 (5)	0.03168 (19)	0.0503 (8)	
O1	0.5081 (2)	0.0924 (4)	0.09705 (15)	0.0718 (9)	
H1A	0.4679	0.0450	0.0471	0.108*	
C12	0.5499 (2)	0.3958 (5)	0.20504 (18)	0.0443 (8)	
C13	0.4735 (3)	0.3145 (5)	0.10995 (18)	0.0471 (8)	
C11	0.5673 (3)	0.2553 (6)	0.2819 (2)	0.0619 (9)	
H11	0.5343	0.1162	0.2752	0.074*	
C6	0.3446 (3)	0.3186 (5)	0.11176 (17)	0.0496 (8)	
C7	0.5985 (3)	0.5994 (5)	0.2167 (2)	0.0569 (9)	
H7	0.5862	0.6945	0.1659	0.068*	
C1	0.2929 (4)	0.1361 (7)	0.1416 (2)	0.0754 (12)	
H1	0.3356	0.0079	0.1585	0.090*	
C5	0.2780 (3)	0.5067 (6)	0.0880 (2)	0.0605 (9)	
H5	0.3123	0.6296	0.0690	0.073*	
C9	0.6848 (3)	0.5280 (7)	0.3798 (2)	0.0707 (11)	
H9	0.7307	0.5718	0.4381	0.085*	
C10	0.6348 (3)	0.3262 (7)	0.3685 (2)	0.0742 (11)	
H10	0.6459	0.2334	0.4199	0.089*	
C8	0.6667 (3)	0.6660 (7)	0.3044 (3)	0.0681 (10)	
H8	0.6999	0.8049	0.3116	0.082*	
C4	0.1615 (3)	0.5167 (8)	0.0917 (3)	0.0861 (14)	
H4	0.1181	0.6442	0.0749	0.103*	
C2	0.1750 (5)	0.1500 (11)	0.1458 (3)	0.0978 (17)	
H2	0.1398	0.0299	0.1660	0.117*	
C3	0.1116 (4)	0.3374 (12)	0.1204 (3)	0.1031 (19)	
H3	0.0335	0.3425	0.1228	0.124*	

### Atomic displacement parameters (Å^2^)

**Table:**

	*U*^11^	*U*^22^	*U*^33^	*U*^12^	*U*^13^	*U*^23^
C14	0.0636 (18)	0.0556 (19)	0.0336 (14)	0.0098 (14)	0.0161 (13)	0.0068 (12)
O1	0.129 (2)	0.0447 (14)	0.0443 (12)	0.0142 (13)	0.0267 (13)	−0.0013 (10)
C12	0.0537 (16)	0.0489 (17)	0.0340 (14)	0.0073 (13)	0.0182 (11)	0.0070 (12)
C13	0.0724 (19)	0.0384 (16)	0.0340 (15)	0.0061 (13)	0.0201 (13)	0.0056 (11)
C11	0.078 (2)	0.062 (2)	0.0448 (17)	−0.0043 (16)	0.0145 (15)	0.0139 (15)
C6	0.0677 (19)	0.0574 (19)	0.0246 (13)	−0.0177 (15)	0.0138 (12)	−0.0078 (12)
C7	0.067 (2)	0.0514 (19)	0.0527 (18)	0.0034 (15)	0.0167 (15)	0.0098 (14)
C1	0.105 (3)	0.076 (2)	0.0483 (19)	−0.031 (2)	0.0263 (18)	−0.0002 (17)
C5	0.063 (2)	0.066 (2)	0.0534 (19)	0.0021 (16)	0.0166 (15)	−0.0072 (16)
C9	0.059 (2)	0.099 (3)	0.0489 (19)	0.004 (2)	0.0057 (15)	−0.0053 (19)
C10	0.080 (2)	0.096 (3)	0.0424 (18)	0.004 (2)	0.0081 (16)	0.0191 (18)
C8	0.067 (2)	0.070 (2)	0.064 (2)	−0.0095 (18)	0.0111 (16)	−0.0126 (18)
C4	0.068 (2)	0.122 (4)	0.068 (2)	0.001 (2)	0.0177 (19)	−0.035 (2)
C2	0.108 (3)	0.132 (4)	0.063 (3)	−0.073 (3)	0.038 (2)	−0.032 (3)
C3	0.081 (3)	0.161 (5)	0.078 (3)	−0.046 (4)	0.039 (2)	−0.052 (3)

### Geometric parameters (Å, °)

**Table:**

C14—C14^i^	1.178 (5)	C1—C2	1.408 (7)
C14—C13	1.480 (4)	C1—H1	0.9300
O1—C13	1.445 (3)	C5—C4	1.389 (5)
O1—H1A	0.8200	C5—H5	0.9300
C12—C7	1.363 (4)	C9—C10	1.359 (5)
C12—C11	1.395 (4)	C9—C8	1.370 (5)
C12—C13	1.541 (4)	C9—H9	0.9300
C13—C6	1.526 (4)	C10—H10	0.9300
C11—C10	1.389 (5)	C8—H8	0.9300
C11—H11	0.9300	C4—C3	1.362 (7)
C6—C5	1.385 (5)	C4—H4	0.9300
C6—C1	1.395 (5)	C2—C3	1.366 (7)
C7—C8	1.396 (5)	C2—H2	0.9300
C7—H7	0.9300	C3—H3	0.9300
			
C14^i^—C14—C13	178.3 (4)	C2—C1—H1	120.6
C13—O1—H1A	109.5	C6—C5—C4	121.8 (4)
C7—C12—C11	119.4 (3)	C6—C5—H5	119.1
C7—C12—C13	122.5 (2)	C4—C5—H5	119.1
C11—C12—C13	118.1 (3)	C10—C9—C8	119.2 (3)
O1—C13—C14	108.4 (2)	C10—C9—H9	120.4
O1—C13—C6	109.5 (3)	C8—C9—H9	120.4
C14—C13—C6	110.7 (2)	C9—C10—C11	121.6 (3)
O1—C13—C12	107.7 (2)	C9—C10—H10	119.2
C14—C13—C12	111.3 (2)	C11—C10—H10	119.2
C6—C13—C12	109.2 (2)	C9—C8—C7	120.4 (3)
C10—C11—C12	119.0 (3)	C9—C8—H8	119.8
C10—C11—H11	120.5	C7—C8—H8	119.8
C12—C11—H11	120.5	C3—C4—C5	119.1 (5)
C5—C6—C1	118.7 (3)	C3—C4—H4	120.5
C5—C6—C13	120.7 (3)	C5—C4—H4	120.5
C1—C6—C13	120.6 (3)	C3—C2—C1	120.9 (4)
C12—C7—C8	120.4 (3)	C3—C2—H2	119.5
C12—C7—H7	119.8	C1—C2—H2	119.5
C8—C7—H7	119.8	C4—C3—C2	120.8 (4)
C6—C1—C2	118.7 (5)	C4—C3—H3	119.6
C6—C1—H1	120.6	C2—C3—H3	119.6
			
C14^i^—C14—C13—O1	7(15)	C12—C13—C6—C1	−90.2 (3)
C14^i^—C14—C13—C6	−114 (15)	C11—C12—C7—C8	1.0 (5)
C14^i^—C14—C13—C12	125 (15)	C13—C12—C7—C8	179.7 (3)
C7—C12—C13—O1	137.1 (3)	C5—C6—C1—C2	0.4 (5)
C11—C12—C13—O1	−44.2 (3)	C13—C6—C1—C2	177.5 (3)
C7—C12—C13—C14	18.4 (4)	C1—C6—C5—C4	−1.0 (5)
C11—C12—C13—C14	−162.8 (3)	C13—C6—C5—C4	−178.1 (3)
C7—C12—C13—C6	−104.1 (3)	C8—C9—C10—C11	1.1 (6)
C11—C12—C13—C6	74.7 (3)	C12—C11—C10—C9	−0.6 (6)
C7—C12—C11—C10	−0.5 (5)	C10—C9—C8—C7	−0.6 (5)
C13—C12—C11—C10	−179.3 (3)	C12—C7—C8—C9	−0.4 (5)
O1—C13—C6—C5	−155.4 (2)	C6—C5—C4—C3	0.6 (5)
C14—C13—C6—C5	−36.0 (4)	C6—C1—C2—C3	0.5 (6)
C12—C13—C6—C5	86.8 (3)	C5—C4—C3—C2	0.4 (6)
O1—C13—C6—C1	27.6 (3)	C1—C2—C3—C4	−0.9 (7)
C14—C13—C6—C1	147.0 (3)		

Symmetry codes: (i) −*x*+1, −*y*+1, −*z*.

### Hydrogen-bond geometry (Å, °)

**Table:**

*D*—H···*A*	*D*—H	H···*A*	*D*···*A*	*D*—H···*A*
O1—H1a···O1^ii^	0.82	2.37	3.040 (3)	139

Symmetry codes: (ii) −*x*+1, −*y*, −*z*.
